# Use of Simvastatin, Fibrin Clots, and Their Combination to Improve Human Ovarian Tissue Grafting for Fertility Restoration After Anti-Cancer Therapy

**DOI:** 10.3389/fonc.2020.598026

**Published:** 2021-01-22

**Authors:** Roei Magen, Yoel Shufaro, Yair Daykan, Galia Oron, Elena Tararashkina, Shulamit Levenberg, Eli Anuka, Avi Ben-Haroush, Benjamin Fisch, Ronit Abir

**Affiliations:** ^1^Infertility and IVF Unit, Beilinson Women Hospital, Rabin Medical Center, Petach Tikvah, Israel; ^2^Goldman Medical School, Faculty of Health Sciences, Ben-Gurion University of the Negev, Beer-Sheva, Israel; ^3^Sackler Faculty of Medicine, Tel Aviv University, Tel Aviv, Israel; ^4^The Felsenstein Medical Research Center, Sackler Faculty of Medicine, Tel-Aviv University, Tel Aviv, Israel; ^5^Faculty of Biomedical Engineering, Technion-Israel Institute of Technology, Haifa, Israel; ^6^Department of Biological Chemistry, The Alexander Silberman Institute of Life Sciences, The Hebrew University of Jerusalem, Jerusalem, Israel

**Keywords:** transplantation, human ovarian tissue, immunodeficient mice, simvastatin, fibrin clots, mouse platelet endothelial cell adhesion molecule, human-specific antibodies for von Willebrand factor, fertility preservation/restoration for cancer patients

## Abstract

Anticancer treatments, particularly chemotherapy, induce ovarian damage and loss of ovarian follicles. There are limited options for fertility restoration, one of which is pre-chemotherapy cryopreservation of ovarian tissue. Transplantation of frozen-thawed human ovarian tissue from cancer survivors has resulted in live-births. There is extensive follicular loss immediately after grafting, probably due to too slow graft revascularization. To avoid this problem, it is important to develop methods to improve ovarian tissue neovascularization. The study’s purpose was to investigate if treatment of murine hosts with simvastatin or/and embedding human ovarian tissue within fibrin clots can improve human ovarian tissue grafting (simvastatin and fibrin clots promote vascularization). There was a significantly higher number of follicles in group A (ungrafted control) than in group B (untreated tissue). Group C (simvastatin-treated hosts) had the highest levels of follicle atresia. Group C had significantly more proliferating follicles (Ki67-stained) than groups B and E (simvastatin-treated hosts and tissue embedded within fibrin clots), group D (tissue embedded within fibrin clots) had significantly more proliferating follicles (Ki67-stained) than group B. On immunofluorescence study, only groups D and E showed vascular structures that expressed both human and murine markers (mouse-specific platelet endothelial cell adhesion molecule, PECAM, and human-specific von Willebrand factor, vWF). Peripheral human vWF expression was significantly higher in group E than group B. Diffuse human vWF expression was significantly higher in groups A and E than groups B and C. When grafts were not embedded in fibrin, there was a significant loss of human vWF expression compared to groups A and E. This protocol may be tested to improve ovarian implantation in cancer survivors.

## Introduction

Major advances in anticancer treatment have resulted in a significant increase in the survival rates of young female patients (1, 2). However, anticancer treatments, particularly chemotherapy, induce ovarian damage and loss of ovarian follicles. Human ovarian cortical tissue contains numerous immature ovarian follicles, and its cryopreservation before chemotherapy is a major option for fertility preservation (1–4).

So far, transplantation of frozen-thawed human ovarian tissue has resulted in over 130 live births in cancer survivors (5). However, there is extensive follicular loss immediately after grafting, probably secondary to too slow graft revascularization, and the resulting apoptosis and ischemia (1, 2). Therefore, it is important to develop novel methods to improve ovarian tissue fusion, regeneration, and neovascularization that do not cause side effects and can be safely applied in auto-transplantation in humans.

Simvastatin inhibits cholesterol biosynthesis and is used as a first-line treatment option for hypercholesterolemia (6). It has been found in animal studies to prevent pregnancy complications such as recurrent abortions, and intrauterine growth retardation (6), and to enhance wound healing and vascularization (7). Using mouse models, researchers either administered the drug orally (in a single injected dose) with and without methylprednisolone (an anti-inflammatory agent) before oophorectomy followed one week later by auto-transplantation of vitrified-warmed murine ovarian portions (8, 9), or injected simvastatin systemically before and after grafting fresh ovarian tissue (10). This treatment protected the ovary against ischemic injury, enhanced neovascularization (visualized by longitudinal magnetic resonance imaging combined with fluorescence markers), increased vascular support to the ovarian grafts, decreased follicular apoptosis (9, 10), and activated the Akt1 signal pathway (responsible for the initial development of primordial follicles) (10, 11). Combining orally administered simvastatin and injected methylprednisolone enhanced the quality of the grafted ovarian mouse tissue, improved follicle survival, and reduced follicular apoptosis (8). In addition, anti-Mullerian hormone was measured in the host’s blood and immature oocytes underwent *in vitro* maturation followed by *in vitro* fertilization and development to blastocysts.

Fibrin clots were among the first biomaterials to prevent bleeding and promote wound healing in humans (12). Additionally, fibrin has been found to promote angiogenesis by inducing growth of new blood vessels from existing vasculature (13, 14). Fibrin clots have also been used to embed isolated mouse follicles for development of an artificial ovary model (15). One study showed the ability of grafted fibrin matrix with multi-cellular constructs to increase vascularization, leading to the creation of vascular networks within the graft itself and after transplantation, between the graft and the host (16).

The aims of the present study were to determine if treating murine hosts with simvastatin or embedding human ovarian tissue in fibrin clots or their combination improves human ovarian tissue grafting outcomes.

## Material and Methods

### Human Ovarian Tissue

Approval for the study was granted by the local institutional ethics committee. Ovarian samples were obtained from six patients with cancer, mean age = 14.5±5 years (only one patient aged 23 years and the rest minors), undergoing laparoscopic ovarian surgery for fertility preservation before the initiation of anti-cancer treatment (**Table I**). There were no statistically significant differences in age between the groups. None of the patients had ovarian diseases. We used similar sized groups in our previous studies (17–20). Informed consent to donate tissue for this study was obtained from the patients or, for minors, from their parents. One slice measuring ~5X5 mm from every ovarian sample was fixed in Bouin’s solution immediately after ovarian excision and before cryopreservation (fresh-ungrafted control) and prepared for histology. This procedure was done to ensure that the grafted tissue contained follicles (17, 18). As the study was conducted with human ovarian tissue, and the donated tissue per patient was very limited, we did not have sufficient tissue amounts from the same patient to allocate to all groups in parallel. In this study as well as in our previous similar ones (17, 18, 20) we attempted to utilize ovarian tissue from young patients all before chemotherapy, mostly similarly aged (**Table I**). In this manner we utilized samples with relatively high follicle numbers. Moreover, the number of hosts in every group was relatively large.

**Table I:** Transplantation of ovarian tissue into immunodeficient mice.

Treatment	Patient		Patient Age(yr)	No. of Hosts
**Group B**		1	13	4
		2	13	5
**Total**				9
**Group C**		3	12	4
		4	16	3
		5	23	7
**Total**				14
**Group D**		1	13	4
		5	23	4
**Total**				8
**Group E**		2	13	5
		6	10	4
**Total**				9

Group B = untreated tissue grafted into untreated hosts, Group C = untreated tissue grafted into hosts treated with simvastatin, Group D = ovarian tissue embedded in fibrin clots, Group E = ovarian tissue embedded in fibrin clots and hosts treated with simvastatin.

### Cryopreservation and Thawing of Ovarian Tissue

**Figure 1:**
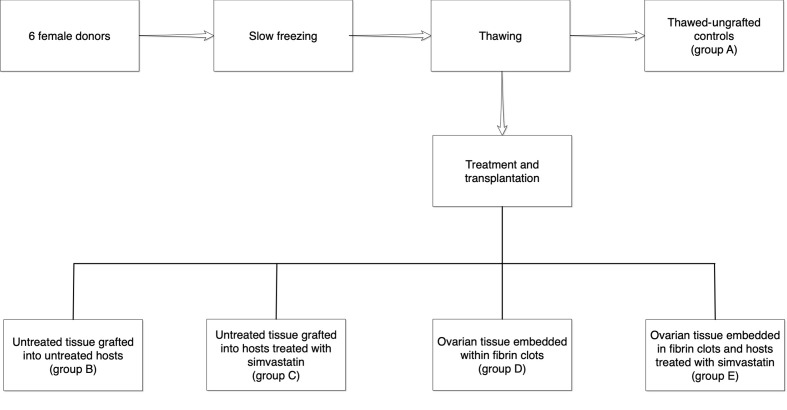
Illustration of the treatment and control groups. One slice from each ovarian sample was fixed immediately for histological evaluation (group A). The remaining thawed samples were then divided into four groups as follows: B = untreated tissue grafted into untreated hosts, C = untreated tissue grafted into hosts treated with simvastatin, D = ovarian tissue embedded within fibrin clots, E = ovarian tissue embedded in fibrin clots and hosts treated with simvastatin.

The ovarian samples were sliced thinly and placed in a 1.5M dimethylsulfoxide (DMSO, Sigma, St. Louis, MO, USA) solution (17, 18, 20) for slow gradual freezing. Samples were then kept on ice for 30 min to allow equilibration, frozen slowly and gradually in a programmable freezer (Kryo 360-1/7, Planer Biomed, Sunbury on Thames, UK) and immediately placed in liquid nitrogen. Thawing was conducted by washouts with decreasing concentration gradients of DMSO (1M, 0.5M, 0M) in phosphate-buffered saline (PBS, Biological industries, Beit-Haemek, Israel) with 20% serum substitute supplement (Irvine Scientific, Santa Ana, CA, USA) at room temperature, with a final incubation at 37^0^C. One slice similar in size to the fresh-ungrafted control was fixed immediately after thawing in Bouin’s solution (group A, thawed-ungrafted controls, **Figure 1**). We compared only this untransplanted control and not the fresh controls to the transplantation groups, as in auto-transplantation clinical studies only frozen-thawed samples are being used.

### Preparation of Fibrin-Thrombin Clots (Hydrogels) With Human Ovarian Tissue

**Figure 2:**
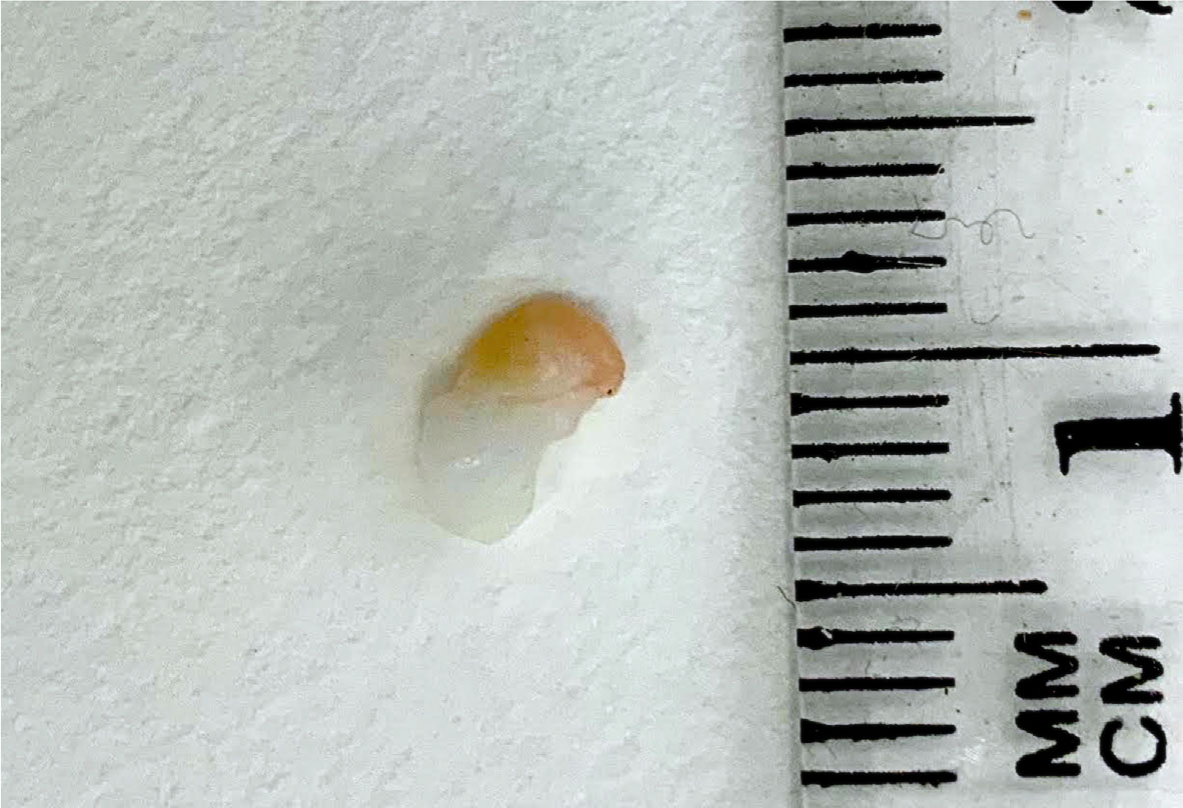
Human ovarian cortical tissue embedded in a fibrin clot before grafting.

Our laboratory has previous successful experience in embedding both human ovarian tissue and isolated follicles in hydrogels (21, 22). Thrombin solutions from human plasma (50 µl, Sigma) were pipetted into small Eppendorf test tubes (Simada, Holon, Israel), and ovarian specimens were carefully placed on the solution (see groups below) and covered with a fibrinogen solution (100 µl, Sigma) from human plasma (16), enabling clotting with fully embedded ovarian tissue (**Figure 2**).

### Transplantation Into Immunodeficient Mice

Approval for this experiment has been obtained from the institutional Animal Supervision and Experimentation Committee. Forty immunodeficient nu/nu female Balb/C mice aged 10–12 weeks (Envigo, Jerusalem, Israel) were used for tissue transplantation (17). From our experience this was a sufficient number to draw clear statistical conclusions (17, 18). The operations were carried out in sterile conditions in special animal facilities under anesthesia (18, 23). The back muscle was used as a transplantation site, as it is not richly vascularized and could mimic ovarian conditions in this regard. After dissecting the back muscle, one ovarian slice per host (measuring ~5X5 mm, similar to the ungrafted controls) was pushed inside, and the opening was sutured. Four experimental groups and one control group were compared (**Figure 1**).

B. Untreated tissue grafted into untreated hosts (untreated grafted controls).

C. Untreated tissue grafted into hosts treated with simvastatin (Teva, Petach-Tikva, Israel) placed in the drinking water (10 mg/liter). This concentration was chosen as at higher concentrations, the mice became abnormally thin. The oral route of administration was chosen because when the procedure is extrapolated to humans, it will be easier to administer the drug in tablet form as opposed to injections (10). Furthermore, in previous studies conducted in our laboratory, the oral route of administration was used for vitamin E or melatonin and yielded satisfactory results (17, 18).

D. Ovarian tissue embedded within fibrin clots (Sigma) and grafted into untreated hosts.

E. Ovarian tissue samples embedded in fibrin clots (Sigma), and grafted into hosts treated with simvastatin (Teva) as in group C.

The hosts were sacrificed after a post-operative period of three weeks and the grafts were removed and fixed.

#### Histological Preparation for Light Microscopy (LM)

Fresh and thawed-ungrafted control samples (group A), and grafted ovarian tissue specimens (groups B–E) were prepared for paraffin (Surgipath Paraplast Plus, Leica Biosystems, Richmond, IL, USA) embedding and hematoxylin (Mayer Hematoxylin, Pioneer Research Chemicals Ltd., Colchester, UK) and eosin (Sigma) staining (17, 18). In all specimens, preantral follicles (atretic and non-atretic) were counted throughout the field (magnification X100) in two levels (with at least 50 µm between levels to avoid counting the same follicle twice) using a computerized image analyzer (analySIS, Soft Imaging System, Digital Solutions for Imaging and Microscopy, System GmbH, Munster, Germany). As we conducted various evaluation methods we were unable to calculate follicle density, as in our previous studies (17, 18, 20).

Atretic follicles were identified by the presence of pyknotic cells, eosinophilia of the ooplasm, and clumping of the chromatin material (3, 17, 24). Unstained sections were placed on OptiPlus positive-charged microscope slides (BioGenex Laboratories, San Ramon, CA, USA) for evaluation of apoptosis using the terminal deoxynucleotidyl transferase (TdT) (TUNEL) assay, for immunohistochemistry (IMH) studies of the neovascularization marker, platelet endothelial cell adhesion molecule (PECAM), and the granulosa cell proliferation marker, Ki67, and for the immunofluorescence (IF) studies of the species-specific neovascularization markers, mouse PECAM and human von Willebrand factor (vWF).

#### TdT Assay (TUNEL)

Apoptosis was evaluated with a TdT assay (ApopTag In Situ Detection Kit; Intergen Company, Purchase, NY) (17–19). The assay was repeated for two sections per case. The slides were deparaffinized, dehydrated, and rinsed. Positive controls were prepared by pretreating samples with DNase I (specific activity 1,000–10,000 U/ml; Sigma). Thereafter, all slides were treated with proteinase K (Sigma). To block endogenous peroxidase activity, the samples were quenched with 3% hydrogen peroxide (Gadot Biochemical Industries Ltd, Haifa Bay, Israel). The slides were then incubated with working-strength TdT at 37°C. The negative controls were incubated with distilled water. Thereafter, samples were incubated with conjugate anti-digoxigenin peroxidase, and exposed to a diaminobenzidine urea H_2_O_2_ solution (brown staining = apoptosis) (DAB Quanto, Thermo Scientific, Cheshire, UK). The sections were then counterstained with Mayer hematoxylin (Pioneer Research Chemicals Ltd). Finally, the slides were rinsed with running tap water and rehydrated. Staining of TUNEL-expressing stroma cells was graded on a 4-point scale according to intensity as described by us in several previous studies (17–19): 0 = no TUNEL staining, 1 = low staining intensity, 2 = medium staining intensity, 3 = high staining intensity. The slides were viewed independently and blindly by three of the authors (RM, YD, and RA).

#### IMH for Ki67 and PECAM

Ki67 is a cell cycle-associated nucleoprotein antigen that serves as a marker of proliferation during the active cell cycle phases (G1, S, G2 and mitosis) (22). An increase in Ki67 staining correlates directly with activation of the flat granulosa cells of primordial follicles to cuboidal granulosa cells as well as with follicular growth.

PECAM is a member of the cell adhesion superfamily of molecules (19, 25), encoded by the PECAM gene on chromosome 17 and expressed in endothelial cells and most blood cells. PECAM molecules function in endothelial intracellular junctions of confluent vascular beds. It serves as a neovascularization marker in ovaries (19, 26), and is the most common marker used to identify changes in blood supply after grafting.

For IMH, sections were first dehydrated. Slides were microwaved with citrate buffer at pH 6.0 (CheMate buffer, DAKOCytomation, Glostrup, Denmark) to enhance antigen retrieval, and quenched in 3% hydrogen peroxide (Gadot Biochemical Industries LTD) to block endogenous peroxidase activity (19, 22). The sections were then incubated with either a rabbit polyclonal anti-Ki67 antibody (ab16667, Abcam, Cambridge, UK) or an anti-PECAM antibody (sc-376764, Santa Cruz Biotechnology, Santa Cruz, CA, USA). For the negative controls, we used a normal rabbit IgG antibody (sc-2027, Santa Cruz Biotechnology). Solutions from a Dako EnVision System, horseradish peroxidase–3-amino-9-ethylcarbazole (AEC) kit (Dako Corporation, Glostrup, Denmark) were used for immunostaining: red-brown staining indicated Ki67 or PECAM expression. We defined a follicle as being positively stained for Ki67 if at least one of its granulosa cells expressed Ki67 (22). PECAM staining intensity was analyzed in the periphery of the graft (representing neovascularization occurring between the graft and the host, peripheral pattern) and in the center of the graft (representing the blood vessel proliferation within the graft of both human and mouse origin, diffuse pattern) (19, 27). For each pattern, staining intensity in PECAM-expressing cells was graded on a 4-point scale as described by us previously (19): 0 = no PECAM staining, 1 = low staining intensity, 2 = medium staining intensity, 3 = high staining intensity. All slides were viewed independently and blindly by three investigators (RM, YD, and RA).

#### IF of vWF and Mouse PECAM for Confocal Laser Microscopy

VWF is a large, multimeric glycoprotein made by endothelial cells. It performs two essential functions in hemostasis: it mediates the adhesion of platelets to subendothelial connective tissue, and it binds blood clotting factor VIII (28). Human and mouse-specific vascularization markers have been used to gain a deeper understanding of the vascular changes and interactions in the graft and between the graft and host tissues (27).

For the present study, the sections were first dehydrated (29). To enhance antigen retrieval, all slides were microwaved with tris ethylenediaminetetraacetic acid (Tris-EDTA) buffer at pH 9.0 (diluted from a X10 concentrated Tris-EDTA buffer at pH 9.0, Novus Biologicals, Littleton, CO, USA). Thereafter, the slides were incubated with the blocking buffer [PBS (Biological Industries) combined with 4% serum substitute supplement (Irvine Scientific), 0.05% tween 20 (Sigma), and 0.3% triton (Sigma)]. This was followed by an overnight incubation with a solution of the two primary antibodies in equal volumes (at a concentration of 1:200): unconjugated goat polyclonal antibody against human vWF (LS-B-2590, LSBio, LifeSpan BioSciences, Inc., Seattle, WA, USA) and rabbit polyclonal antibody against mouse PECAM (ab124432, Abcam), diluted in the blocking buffer. The following morning, the sections were further incubated with a solution of the respective secondary antibodies diluted in equal volumes, namely Alexa Fluor 488-conjugated donkey anti goat antibodies (705-545-147, AffiniPure, Jackson Laboratories) and biotin-SP conjugated donkey anti rabbit antibodies (711-065-152, AffiniPure, Jackson Laboratories), diluted with the blocking buffer (all at a concentration of 1: 200). The negative controls included sections incubated only with the diluted secondary antibody solutions without the primary antibodies. To increase Cy3 staining, we further incubated the slides with Cy3-conjugated streptavidin (016-160-084, Jackson Laboratories) diluted (1:100) with PBS (Biological Industries). Finally, the samples were incubated with 4’, 6-diamidino-2-phenylindole (DAPI) background staining (D9564, Sigma) diluted (1:1000) with distilled water. The slides were mounted with a water-based mounting medium (Fluoromount, Diagnostic BioSystems, Pleasanton, CA, USA). Visualization and photography were performed with a Leica SP8 (Leica Microsystems, Wetzlar, Germany) confocal laser microscope. The unspecific very low fluorescence observed in the negative controls was removed from all the IF-stained sections. For consistency, marker-expressing cells in IF sections were graded for staining intensity on a four-point scale, in the same manner as the IMH PECAM-stained sections (19): 0 = no marker expression, 1 = low staining intensity, 2 = medium staining intensity, 3 = high staining intensity. All slides were viewed independently and blindly by three investigators (RM, ET, and RA).

### Statistical Analysis

Apoptosis and vascularization (peripheral and diffuse) grades and follicular count data (including atretic follicles) were analyzed with Kruskal-Wallis one-way analysis of variance on Ranks, followed by Dunn-Bonferroni multiple comparison procedure. Count data for atretic and non-atretic follicles (before and after grafting) and Ki67-stained and unstained follicles (before and after grafting) were analyzed by Chi square test or Fisher’s exact test as indicated, for all pairwise groups. Differences were considered statistically significant when the *P*-value was 0.05. Statistical analysis and graphs were made by using Statistical Package for the Social Sciences (SPSS, IBM, Armonk, NY, USA).

## Results

**Figure 3:**
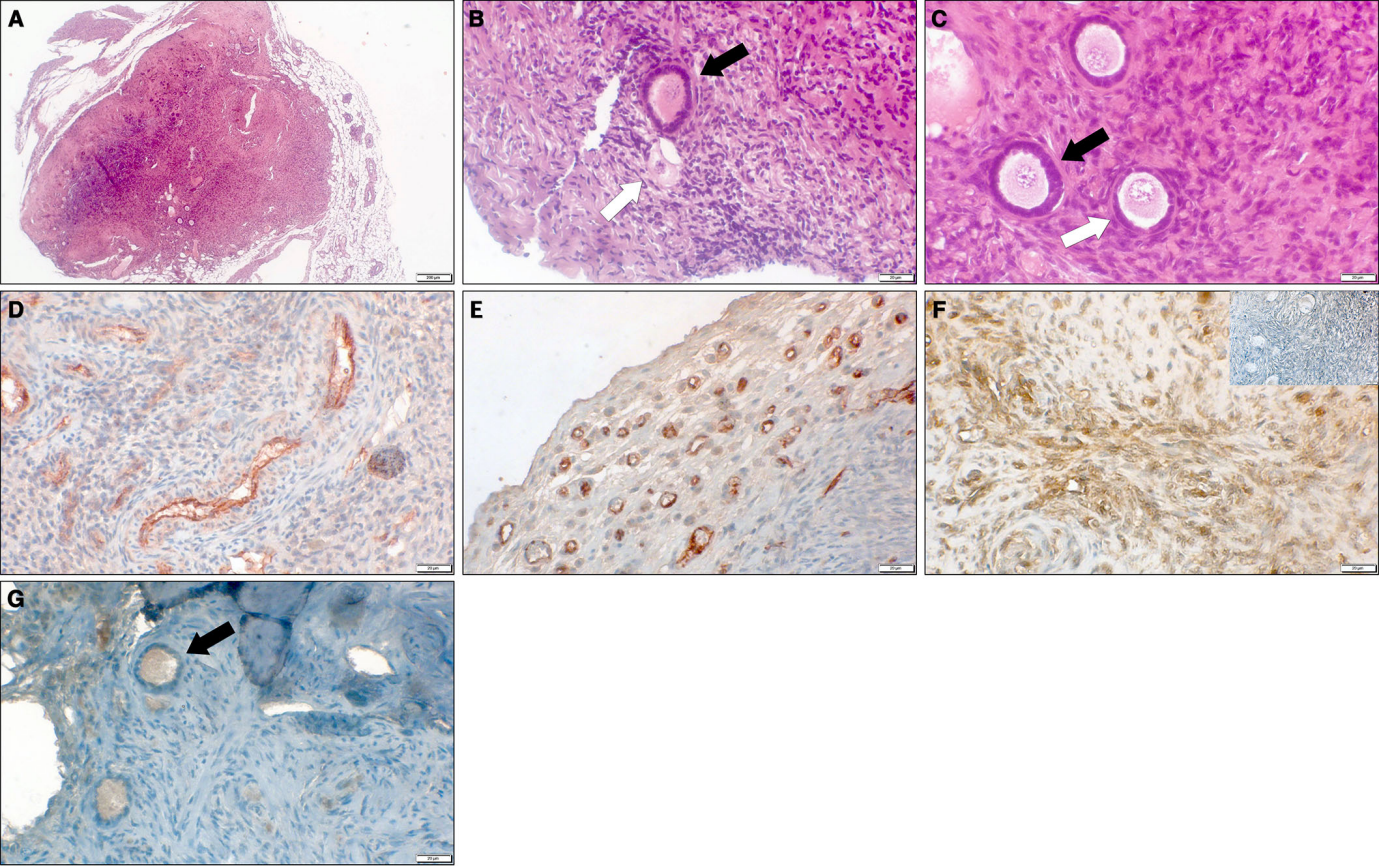
Representative microscopy images of thawed-ungrafted controls and grafts. **(A)** Section of grafted ovarian tissue from a 10-year-old girl, embedded in a fibrin clot combined with host treatment with simvastatin (group E). Note the clear bordered fibrin capsule surrounding the graft. Hematoxlin and eosin, original magnification X100. **(B)** Section of thawed-ungrafted ovarian tissue (group A) from a 13-year-old girl. Note the primary follicle (black arrow) and the primordial atretic follicle (white arrow). Hematoxylin and eosin, original magnification X400. **(C)** Section of grafted ovarian tissue from the same patient as in panel 3A, embedded in a fibrin clot combined with host treatment with simvastatin (group E). Note the primary follicle (black arrow) and primordial follicle (white arrow). Hematoxlin and eosin, original magnification X400. **(D)** Section of thawed-ungrafted ovarian tissue (group A) from a 23-year-old woman. Note the red-brown PECAM staining surrounding the blood vessels in the center of the graft. Original magnification X400. **(E)** Section of grafted ovarian tissue from the same patient as in panel 3D with host treatment with simvastatin (group C). Note the red-brown PECAM staining in the periphery of the graft. Original magnification X400. **(F)** Section of untreated-grafted ovarian tissue (group B) from a 13-year-old girl. Note the brown TUNEL staining surrounding the blood vessels indicative of apoptosis. Note the negative control on the upper right-hand side showing blue staining and lacking brown staining. Original magnification X400. **(G)** Section of grafted ovarian tissue from the same patient as in panel 3A and 3C, embedded in a fibrin clot combined with host treatment with simvastatin (group E). Note the follicle (black arrow) containing red-brown-stained granulosa cells (positive for Ki67) indicating follicular proliferation. Original magnification X400.

All grafts were recovered successfully in all implanted groups. Grafts from groups D (ovarian tissue embedded within fibrin clots) and E (ovarian tissue embedded in fibrin clots and hosts treated with simvastatin) retained their original size and had clear borders. By contrast, grafts from groups B (untreated tissue grafted into untreated hosts) and C (untreated tissue grafted into hosts treated with simvastatin) were difficult to identify within the host muscle and seemed smaller than the samples initially implanted. Morphological images of the ovarian tissue and follicles are shown in **Figure 3**.

**Figure 4:**
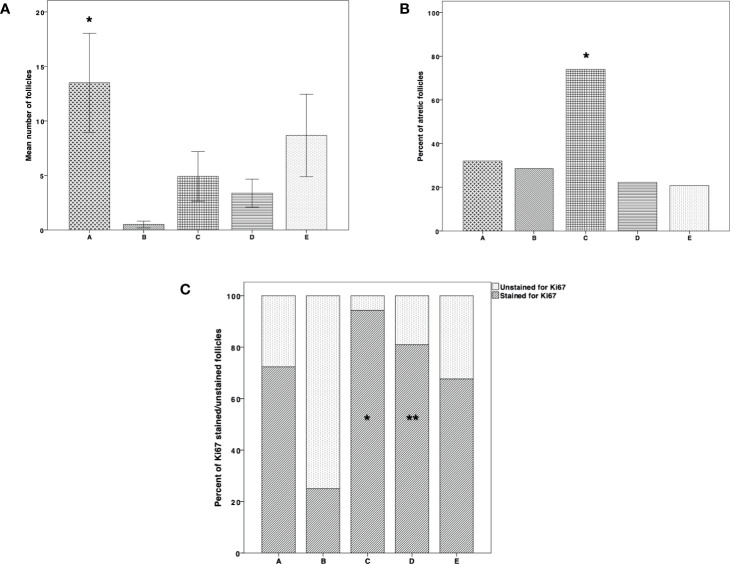
Follicle count, atresia, and proliferation. **(A)** Follicle count in thawed-ungrafted controls and the implanted groups. Results are represented as mean±standard deviation (SD). The *x*-axis represents the five experimental and control groups. The *y*-axis represents the mean number of follicles. Black cross bar = thawed-ungrafted controls (group A). Black diagonal line bar = untreated grafted controls (group B). Black square bar = host treatment with simvastatin (group C). Black horizontal line bar = graft embedding within fibrin clots (group D). Black dot bar = host treatment with simvastatin+graft embedding within fibrin clots (group E). *Significantly higher than group B (*P* = 0.003). **(B)** Follicle atresia in thawed-ungrafted controls and implanted groups. Results are represented as percentage. The *x*-axis represents the five experimental and control groups. The *y*-axis represents the percent of atretic follicles from the total follicles counted. *Significantly higher than in groups A–E (*P* < 0.001, *P* = 0.026, *P* < 0.001, *P* < 0.001, respectively). **(C)** Follicle proliferation in thawed-ungrafted controls and implant groups as demonstrated by Ki67 marker staining. Results are represented as percentage of Ki67 stained and unstained follicles. The *x*-axis represents the five experimental and control groups. The *y*-axis represents the percent of follicles stained and unstained for Ki67 from the total number of follicles counted. Black diagonal line part of bar = percent of Ki67 positively stained follicles. Black dot part of bar = percent of Ki67 unstained follicles. *Significantly higher than in groups B (*P* = 0.004) and E (*P* = 0.002). **Significantly higher than in group B (*P* = 0.05).

**Figure 4A** shows follicle counts in the tissue samples. They were significantly higher in group A (thawed-ungrafted controls) than in group B (P = 0.003). Follicle atresia was significantly higher in group C than in groups B, D, E (*P* < 0.001, *P* = 0.026, *P* < 0.001, *P* < 0.001, respectively) (**Figure 4B**). The number of Ki67 stained follicles was significantly higher in group C than in groups B (*P* = 0.004) and E (*P* = 0.002) and significantly higher in group D than in group B (*P* = 0.05) (**Figure 4C**).

**Figure 5:**
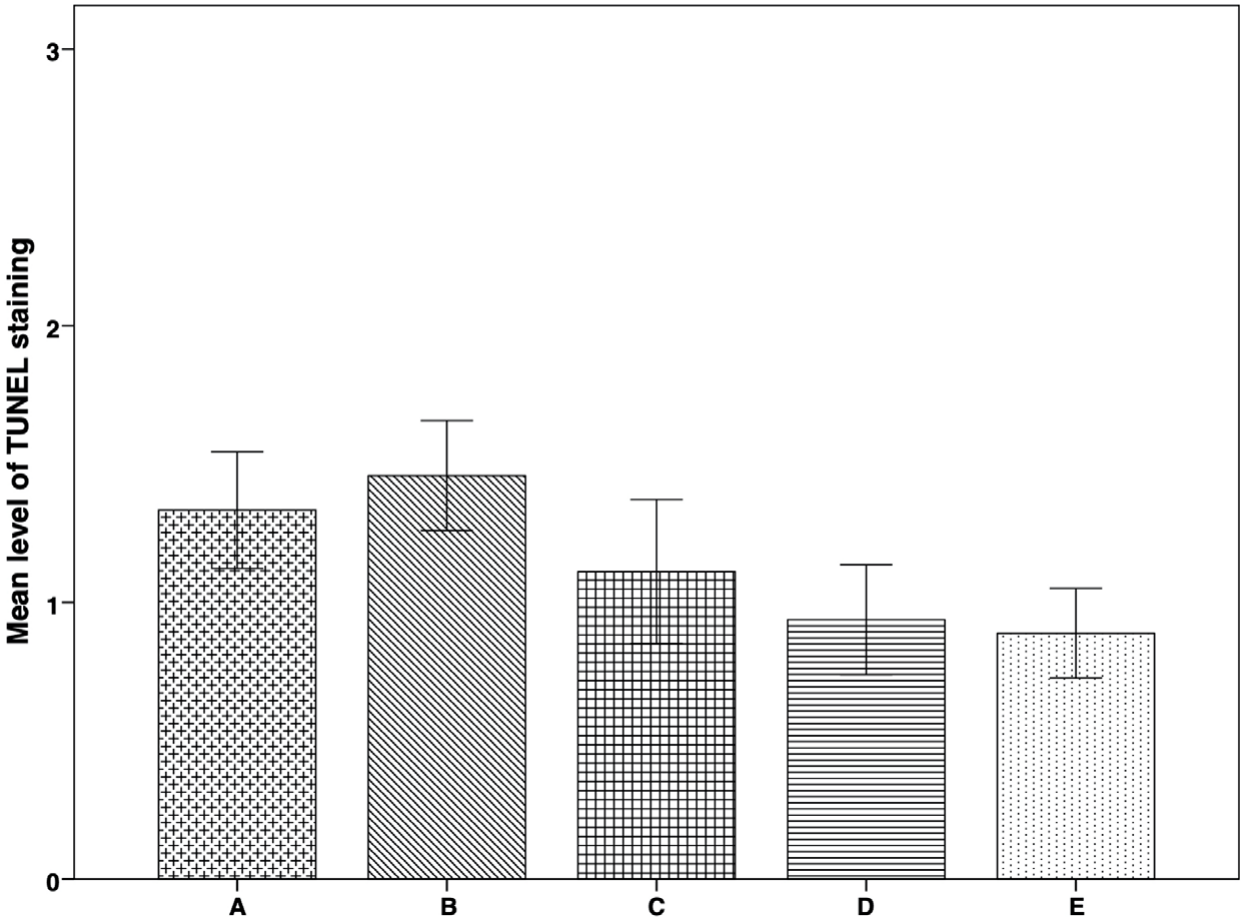
Apoptosis expression in thawed-ungrafted controls and implanted groups. Results are represented as mean+SD. The *x*-axis represents the five experimental and control groups. The *y*-axis represents the level of TUNEL staining. Apoptosis Levels were graded according to staining-intensity: 0 = no TUNEL staining. 1 = TUNEL-expressing cells with low staining intensity. 2 = TUNEL-expressing cells with medium staining intensity. 3 = TUNEL-expressing cells with high staining intensity.

There were no significant differences among the groups in stroma apoptosis levels, demonstrated by the TUNEL staining procedure (**Figure 5**). However, there was a trend of apoptosis increase in group B compared to groups A, C, D, E, although apoptosis levels were also higher in group A. At the same time, the lowest apoptosis levels were identified in groups D and E (**Figures 3F** and **5**). We did not identify any apoptosis in follicles.

**Figure 6:**
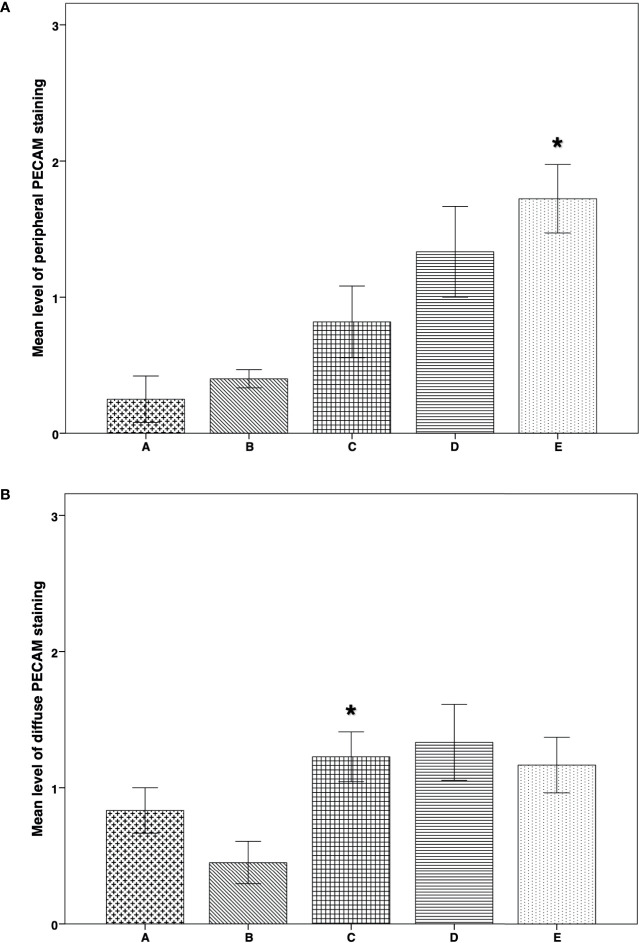
IMH PECAM expression in thawed-ungrafted controls and implant groups. Results are represented as mean+SD. The *x*-axis represents the five experimental and control groups. The *y*-axis represents the level of PECAM staining. **(A)** Peripheral PECAM expression in thawed-ungrafted controls and implant groups. Levels were graded according to staining intensity similar to the TUNEL staining in **Figure 5**. *significantly higher than group A and group B (*P* = 0.005, *P* = 0.008 respectively). **(B)** Diffuse PECAM expression in thawed-ungrafted controls and implant groups. Levels were graded as in **Figures 5** and **6A**.*significantly higher than group B (*P* = 0.045).

IMH study of PECAM expression revealed significantly higher peripheral vascularization in group E than in groups A (*P* = 0.005) and B (*P* = 0.008) (**Figure 6A**). Diffuse vascularization was significantly higher in group C than in group B (*P* = 0.045) (**Figure 6B**). Morphological images of the IMH PECAM assay are shown in **Figures 3D, E**.

**Figure 7:**
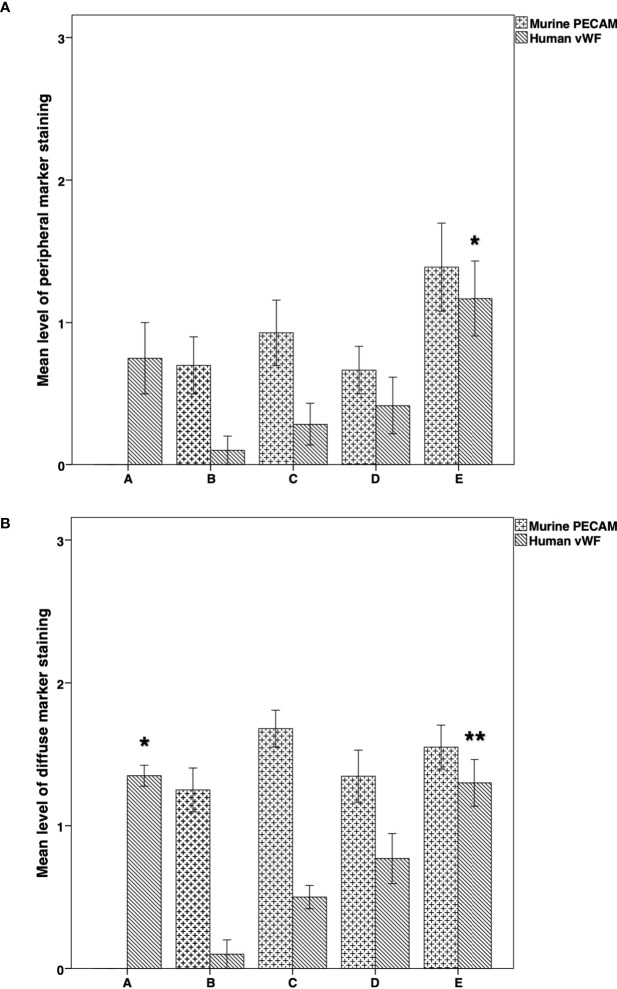
Immunofluorescence expression of mouse-specific PECAM and human- specific vWF in thawed-ungrafted controls and implant groups. Results are represented as mean+SD. The *x*-axis represents the five experimental and control groups. The *y*-axis represents the level of fluorescence marker expression. **(A)** Peripheral mouse-specific PECAM and human-specific vWF expression in thawed-ungrafted controls and grafts. Levels were graded according to the intensity of fluorescence marker expression as in **Figures 5** and **6**. *Significantly higher than group B (*P* = 0.038). **(B)** Diffuse mouse specific PECAM and human specific vWF expression in thawed-ungrafted controls and implanted groups. Levels were graded according to the intensity of fluorescence marker expression as in **Figures 5** and **6**. *Significantly higher than groups B and C (*P* < 0.001 for both comparisons). **Significantly higher than groups B and C (*P* < 0.001, *P* = 0.002, respectively).

**Figure 8:**
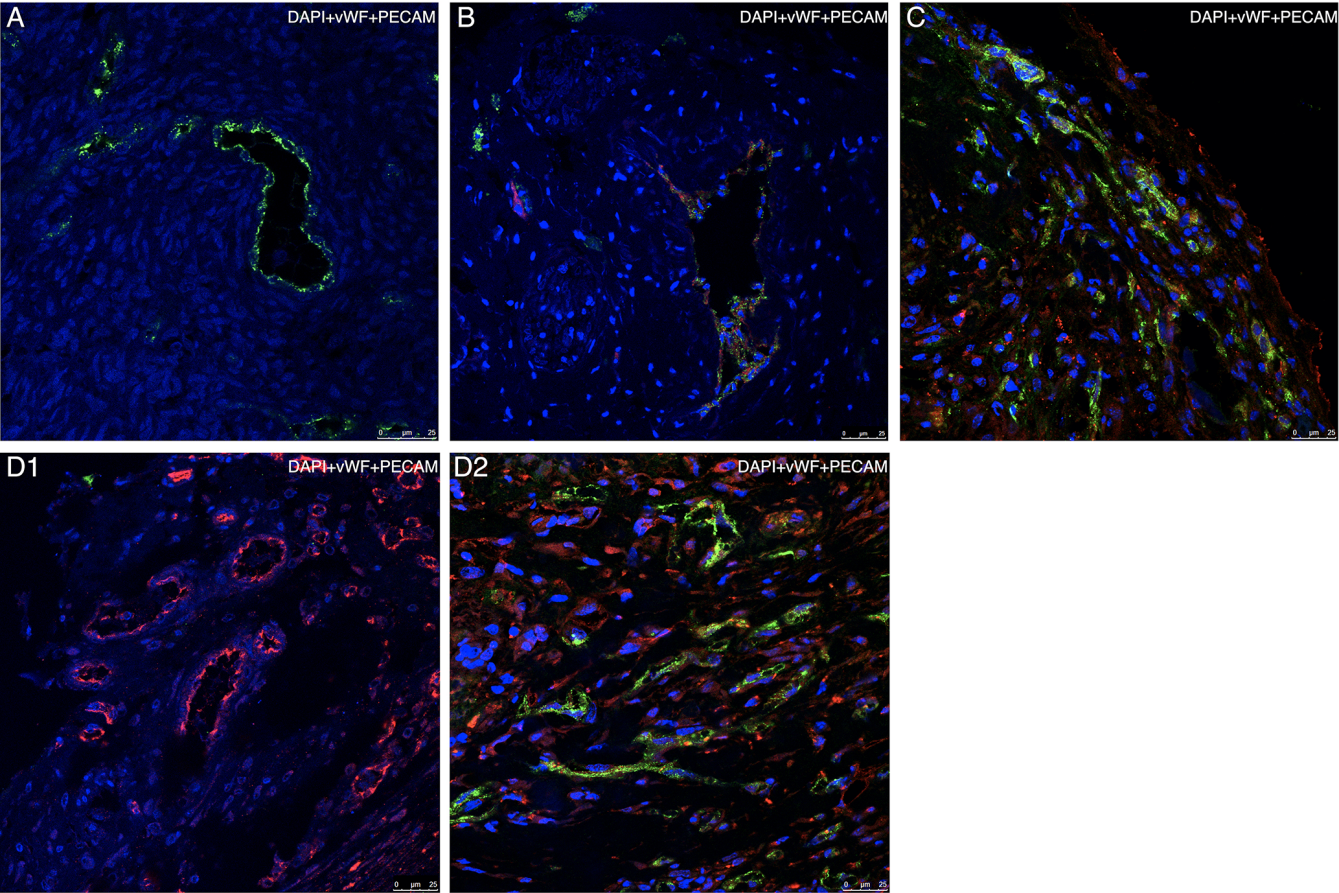
Representative confocal microscopy images of specimens using species- specific immunofluorescence markers of vascularization Each specimen is presented as a merged image compiled of three layers, each representing a separate marker (blue = DAPI, green = human vWF, red = mouse PECAM). **(A)** Section of thawed-ungrafted ovarian sample (group A) from the same patient as in **Figures 3A, C, G**. Note the expression of human vWF markers (green) and lack of mouse PECAM marker expression (red). **(B)** Section of grafted ovarian tissue from the same patient as in **Figures 3D, E**, embedded within a fibrin clot (group D). Note the formation of a large, mixed cell-origin blood vessel expressing both human vWF marker (green) and mouse PECAM marker (red). **(C)** Section of grafted ovarian tissue from the same patient as in **Figures 3A, C, G**, embedded within a fibrin clot and implanted into hosts treated with simvastatin (group E). Note the vascularization occurring in the periphery of the graft originating from both human (green) and mouse (red) origin. **(D1)** Section of untreated-grafted ovarian tissue (group B) from the same patient as in **Figure 3F**. Note the presence of vascular structures originating from mouse origin and lack of vascular structures originating from human origin. **(D2)** Section of grafted ovarian tissue from the same patient as in **Figures 3A, C, G**, embedded within a fibrin clot and implanted into hosts treated with simvastatin (group E). Note, the presence of vascular structures originating from both mouse and human origin (by contrast to image **D1**). Human vasculature loss was reduced when combined treatment (as in group E) was administered.

On examination of the species-specific IF vascularization markers, we found no expression of murine specific markers in any of the stained samples of group A (**Figures 7**, **8A**). The intensity of the peripheral human vWF expression was significantly higher in group E than in group B (*P* = 0.0**38**) (**Figure 7A**). Similarly, the intensity of the diffuse human vWF expression was significantly higher in group E than in groups B (*P* < 0.001) and C (*P* = 0.002). It was also significantly higher in group A than in groups B and C (*P* <0.001 for both comparisons) (**Figure 7B**).

Comparison of the murine and human markers showed that within groups B and C the intensity of the peripheral murine PECAM expression in the two groups was significantly higher than the intensity of human vWF (*P* = 0.03) (**Figure 7A**). Within groups B–D, the intensity of diffuse murine PECAM expression was significantly higher than human vWF intensity expression (*P* < 0.001, *P* < 0.001, *P* = 0.046, respectively) (**Figure 7B**). Moreover, although all grafted specimens showed some areas expressing both human and murine markers, only in specimens from groups D and E we did identify large, organized, vascular structures that expressed both human and murine markers (**Figure 8B**). Morphological images of the IF assay are shown in **Figure 8**. No significant differences were found for PECAM staining patterns between IMH and IF within individual groups.

## Discussion

The present study examined for the first time the effects of treating hosts with simvastatin, embedding ovarian grafts in fibrin clots, and their combination to improve human ovarian tissue implantation for putative fertility restoration in cancer patients. Comparison among four experimental groups and one control group yielded several important findings. Only in the groups in which the grafts were embedded within fibrin clots, either alone (group D) or combined with host treatment with simvastatin (group E) did the implants retain their original size. Follicle number was significantly higher in ungrafted samples than in the untreated group (group B). Treatment of the host alone (group C) was associated with the highest level of follicular atresia compared to the other grafting conditions (groups B, D, E) and their respective controls, but it also led to significantly higher levels of proliferation, as indicated by Ki67 staining than when no treatment was used (group B) or host treatment was combined with graft treatment (group E). There were no significant differences in apoptosis levels among the groups; however, levels were highest in the untreated grafted controls and lowest when the grafts were embedded in fibrin clots (groups D and E). Group D also had a significantly higher number of Ki67-stained follicles than group B. PECAM peripheral expression, one marker of neovascularization, was significantly elevated in the combined treatment (group E) than in the ungrafted and untreated groups (groups A and B, respectively), and the level of diffuse neovascularization was significantly higher in the host-only treated group (group C) than in the untreated group (group B). The groups in which the tissue was embedded in fibrin clots (groups D and E) showed the formation of large, organized, vascular structures expressing both human and murine markers. On IF confocal microscopy studies to determine if the vascularization was of human or mouse origin, we found a significantly higher expression of peripheral human vWF in the combined group (group E) than in the untreated control group (group B). Peripheral murine PECAM expression in group B was significantly higher than the intensity of human vWF. Diffuse human vWF expression was significantly higher in the ungrafted control samples and the combined-treatment group (groups A and E) than in the untreated and host-only treated groups (groups B and C). In the untreated, host-only treated and graft-only treated groups, the level of diffuse murine PECAM marker expression was significantly higher than the level of human vWF marker expression. No significant differences in results were found between the IMH and IF PECAM assays.

The significantly high proliferation rate combined with the significantly high level of follicular atresia found after host treatment with simvastatin (group C) may be explained by the “burn-out effect” demonstrated in an earlier study of grafting of bovine, marmoset, and human ovarian tissue (30, 31). The authors found that implantation resulted in vast follicular recruitment leading to high granulosa cell proliferation (demonstrated by Ki67 expression) followed by follicular depletion and loss. This effect was observed as early as three days after grafting. The process was associated with the activation of the PI3K/PTEN/Akt signaling pathway, which is known to stimulate normal early follicle activation (11). As simvastatin is itself an activator of the PI3K/Akt pathway (32), it could have caused “burn-out” in group C. Furthermore, the PI3K/Akt pathway was also shown to induce anti-apoptotic effects, increase endothelial cell survival and endothelial nitric oxide synthase activity, activate angiogenesis, and act as a mediator of angiogenic processes (32). Its effects on angiogenesis may explain the increased vascularization seen in the simvastatin-treated groups. Accordingly, a previous study in a mouse model found that simvastatin administration reduced ischemic and reperfusion damage to cardiac tissue by increasing the expression of PI3K/Akt pathway, which in turn further increased the expression of endothelial nitric oxide synthase and led to improved tissue health and function (33). In the present study, when simvastatin was combined with graft embedding within fibrin clots, its negative effects (promoting follicular atresia) might have been reversed by the fibrin clot’s promotion of vascularization (13, 14, 16). It is also possible that the fibrin clots acted as shielding agents, protecting the follicles inside the graft and maintaining graft vascularization, leaving simvastatin to exert positive effects on vascularization in the graft’s periphery.

The combination of simvastatin’s ability to induce neovascularization and fibrin’s ability to enhance the creation of vascular networks would also explain the significantly higher peripheral IMH PECAM expression seen in the combined-treatment group than in the ungrafted tissue and untreated controls (8, 10, 16, 32). We assume that treatment with both these agents led to a synergistic effect in the graft’s periphery, resulting in increased vascular proliferation and interaction between the host and graft vasculature. Indeed, a previous study (34), found that Akt1 activation increased integrin expression which in turn led to increased binding of endothelial cells and fibrin and improved wound recovery. Thus, it is possible that by activating the PI3K/Akt1 pathway (32), simvastatin increased binding of endothelial cells to fibrin, making it possible for fibrin to more efficiently affect neovascularization.

It is unclear why the level of the diffuse PECAM expression by IMH was significantly higher compared to no treatment when simvastatin alone was used, but not when simvastatin+fibrin clots were used. It is possible that while fibrin clots work in synergism with simvastatin to mediate the creation of vascular networks in the graft’s periphery, they also moderate simvastatin’s ability to affect the vasculature in the center of the graft. This is also in line with the reason why fibrin clots reversed simvastatin’s negative effects on the follicles, as mentioned above.

The lack of differences in PECAM staining patterns by IMH and IF studies indicates that both represent the same vascular processes that occur only in murine hosts. There was no expression of murine-specific markers in the ungrafted control specimens, proving the reliability of the species-specific markers. The finding that the presence of large, organized, vascular structures expressing both human and murine markers was limited to the groups in which the tissue was embedded in fibrin clots, may be attributable to the ability of fibrin to enhance the creation of vascular networks between graft and host after transplantation (16).

The significantly higher diffuse vWF expression in the ungrafted samples than in the untreated group and the host-only treatment group can be explained by a large loss of human vascularization in groups B and C after transplantation. This loss was not seen in the transplant groups in which the graft was embedded in fibrin clots. It is unclear if embedding the grafts prevented the blood-vessel loss or if the loss was compensated by fibrin-induced angiogenesis from the existing vasculature (13, 14). Moreover, in the group that received combined host and graft treatment, the synergistic effect of simvastatin with fibrin clots resulted in significantly higher human vWF marker expression than in groups B and C. The same trend was observed in group B in which the level of the murine peripheral PECAM marker expression was significantly higher than human peripheral vWF expression, and higher for the same comparison in group C.

None of the between-group differences in stroma-cell apoptosis reached statistical significance, although there was a clear trend of higher apoptosis levels in the untreated group than the other transplant groups and the ungrafted controls; the lowest levels were found when the graft was embedded in fibrin clots. We assume that the differences might have been significant had the groups been larger. Be that as it may, in previous studies from our laboratory with similar group sizes, apoptosis was significantly reduced after host and graft treatment with other substances (18, 19). The difference from our study might be due to our utilization of other ovarian samples in the present study than in the older ones.

Previous studies have examined the effects of simvastatin on mouse ovarian tissue grafting (8–10). The simvastatin-induced follicular atresia seen in human ovarian tissue is contradictory to mouse studies which demonstrated a reduction in follicle atresia (8–10). However, these mouse studies used fresh (10) or vitrified-warmed murine ovarian tissue (8, 9) whereas we used slow-frozen-thawed human ovarian tissue. At present, slow-freezing is the method of choice for human ovarian tissue, and there are no conclusive reports of vitrification as the optimal method (2, 19). Furthermore, past studies used systemic (10) and oral (8, 9) simvastatin injections to treat the murine hosts, whereas in our present study, simvastatin was digested by the murine hosts through their drinking water. Although these methodological differences might account for the differences among the studies in follicle atresia (8–10), it may be more logical to assume that the differences in the effects of simvastatin between mice (8–10) and humans (the present study) are species-dependent. In addition, in mice, simvastatin activated the PI3K/Akt pathway (10), which is associated with early physiological follicle activation (11). It is possible that in the mice, simvastatin also promoted neovascularization through the PI3K/Akt pathway (10) without accelerated granulosa cell proliferation, resulting in rapid atresia as shown for human tissue.

Our findings of significantly higher PECAM staining levels by IMH (both peripheral and diffuse) in the simvastatin-treated groups than in the untreated group are in line with previous studies using murine ovarian grafts (8–10). These authors reported that simvastatin treatment was associated with increased graft vascularization as evaluated by a similar IMH PECAM assay to ours (8, 9), as well as by other vascular immunofluorescence markers combined with longitudinal magnetic resonance imaging techniques (10). The increased neovascularization when the ovarian tissue was embedded within fibrin clots is in line with other studies that showed increased graft vascularization when various cells were embedded in a fibrin matrix and implanted in murine hosts (16).

Van Eyck et al. used the same species-specific vascular IF markers as applied here to examine the revascularization after transplantation of frozen-thawed human ovarian tissue into murine hosts (27). However, our study differs from theirs methodologically as we compared groups treated by different protocols and the IF markers served as an auxiliary tool. It is also noteworthy that our ungrafted control tissues were frozen-thawed, while Van Eyck et al. (27) used fresh ungrafted controls. We assumed that because frozen-thawed human ovarian tissue was used for implantation, we should use frozen-thawed samples for controls. Moreover, in the present study, the vascular processes were analyzed at one time point at termination of the experiment, and not at three time points (3, 5, and 10 days), as in the earlier study (27).

In conclusion, the present study of human ovarian tissue grafting into mice showed that the combination of host treatment with simvastatin and graft embedding in fibrin clots resulted in a synergistic effect which led to improved post-implantation outcomes. The use of species-specific vascular immunofluorescence markers detected a significant loss of human vasculature post-transplantation in groups in which ovarian tissue was not embedded within fibrin clots before grafting. Thus, our study suggested that two vascular processes need to be addressed in order to improve human ovarian tissue grafting outcomes for cancer survivors: post-transplantation vascular loss within the graft and neovascularization between the graft and host. It has been shown that pre-existing vasculature in the graft plays crucial roles in the formation of new blood vessels and graft reperfusion (27). It might, therefore, be worthwhile in future studies to focus on reducing post-transplantation graft vasculature loss in order to achieve improved graft neovascularization and reperfusion outcomes. It is possible that the combined protocol of host treatment with simvastatin and graft embedding in fibrin clots may also be tested putatively to improve implantation in clinical ovarian transplantations to former cancer patients. Moreover, novel methods to improve ovarian tissue grafting with various pharmacological agents are being investigated (2, 20) with the objective of increasing post-implantation follicle viability and reducing follicle loss. In future studies, fibrin clots can be supplemented during preparation with pharmacological agents supporting ovarian implantation, thus serving as an efficient vehicle for delivery of these substances to the graft to further improve human ovarian implantation outcomes (12).

## Author’s Note

RM conducted the study as part of the requirements for his MD degree from the Goldman Medical School at the Faculty of Health Sciences, Ben-Gurion University of the Negev.

## Data Availability Statement

The raw data supporting the conclusions of this article will be made available by the authors, without undue reservation.

## Ethics Statement

The studies involving human participants were reviewed and approved by Rabin Medical Center institutional ethics committee. Written informed consent to donate tissue for this study was obtained from the patients or, for minors, from their parents. The animal study was reviewed and approved by Rabin Medical Center Animal Supervision and Experimentation Committee.

## Author Contributions

RM conducted almost all the laboratory work, conducted most of the statistical analysis and wrote most of manuscript. YS assisted in designing the study, assisted in the various drafts of the manuscript, the analysis of the results, and approved its final version. YD conducted some of the laboratory work, wrote parts of the study and approved its final version. GO provided some of the ovarian samples, conducted the transplantations, assisted in the various drafts of the manuscript, and approved its final version. ET conducted the immunofluorescent studies. SL suggested the use of fibrin clots, assisted in their use, assisted in the design of the part of the study with fibrin clots, assisted in the various drafts of the manuscript, and approved its final version. EA assisted with the immunofluorescent studies and protocol, assisted in the various drafts of the manuscript, and approved its final version. AB-H provided some of the ovarian samples and reviewed the final draft of the manuscript. BF assisted in designing the study, assisted in the various drafts of the manuscript, the analysis of the results and approved its final version. RA designed the study and collected the data, assisted in the operations, the IMH and IF studies and viewed the sections, conducted some of the statistical analysis and supervised the writing of the manuscript. All authors contributed to the article and approved the submitted version.

## Conflict of Interest

The authors declare that the research was conducted in the absence of any commercial or financial relationships that could be construed as a potential conflict of interest.
